# Rubeola, Morbilli, or Measles

**Published:** 1838-10-24

**Authors:** N. Chapman

**Affiliations:** Professor of the Theory and Practice of Physic, in the University of Pennsylvania


### THE MEDICAL EXAMINER.

DEVOTED TO MEDICINE, SURGERY, AND THE COLLATERAL SCIENCES.

EDITED BY J. B. BIDDLE, M.D., M. CLYMER, M.D., AND W. W. GERHARD, M.D.

No. 22.] PHILADELPHIA, WEDNESDAY, OCTOBER 24, 1838. [Vol. I.

### RUBEOLA, MORBILLI, OR MEASLES.

*By* N. Chapman, M. D., *Professor of the Theory and Practice of Physic, in the University of Pennsylvania.*

[Reported for this Journal.]

The first of these titles, or rubeola, was formerly written rubiola, or rubiolo, it being derived from the Spanish *rubio*, and came to be changed to *rubeola*, as directly proceeding from the Latin *rubeo*, to be red, or to blush. Morbilli, or morbillo, is a term also of Spanish origin, being a diminutive of *morbo*, and was employed to designate the disease as a lesser degree of small-pox, the greater malady, with which it had been confounded. Meaning mottled, or speckled, the term measles, which is an old English word, was obviously applied to the affection from its presenting such an appearance.

This disease was brought into Europe at the same time, and probably from the same place, as small-pox, and ran so nearly the same course, that it was supposed, as already intimated, to be merely a modification of the latter, and as such, is described by the Arabian writers, who were the first to notice it. As late, indeed, as the period of Diembrenbroeck and Morton, both of whom have given accounts of it, the notion of the identity of the two diseases was still entertained. To Sydenham, the contemporary of these writers, we are indebted for the earliest accurate and discriminating history of measles, and to which, excepting the later contributions of Watson, who wrote in 1763, and the still more recent inquiries of Willan, by each of whom a supposed variety or modification of the disease is pointed out, nothing scarcely has been supplied.

The more common form of measles is entitled *rubeola vulgaris*, and which is usually ushered in by alternate chilliness and heats, languor, pains in the loins and limbs, soon succeeded, in many instances, by anorexia, thirst, sickness of stomach, or vomitings, with whitish tongue, fulness or aching of the head, cough, or rather defluxions from the nostrils and eyes, which latter are somewhat swollen and red,—in the whole resembling the incipiency of catarrh, complicated by gastric irritation. These symptoms, in different cases, vary in the order of precedence, and in degree, and combination. Either the gastric, or pulmonary, may be anticipatory, or the two simultaneously burst forth, or the one preponderate, or an equality exist between them,—and in other respects, there are diversities.

Nearly from the commencement, the fever which quickly follows, is usually considerable,—though at times, for the second or third day light, becoming higher,—and is particularly marked by an increase of heaviness and somnolency before the eruption, which mostly appears about the fourth day. Cases, however, are recorded, and some I have met with, of its taking place with little premonition,—others, in twelve or twenty-four hours, and many of its being postponed to the sixth or seventh day. Twice I have seen it appear on the tenth day, the fever having distinctly intermitted, and Buckholtz has reported a case where it was delayed till the twenty-first day.

The eruption is primarily displayed on the face and neck, where it is always most prominent,—and successively, on the different parts of the body. It comes out in small reddish spots, distinct, circular, a little elevated, and more florid in the centre than the edges. These gradually enlarge, and by a confluence, or running into each other, form patches of a crescentic or semi-lunar shape, with spaces between them, where the skin preserves its natural colour. In the middle of some of these maculæ a slight vesicle is occasionally observable, and I have known the eruption in several instances to approach in aspect an imperfect varicella. Generally, on the trunk and extremities, it differs from that on the face, in being merely an efflorescence in patches without any elevation. It also may partially come out as welts, resembling the inflictions of the rod or whip, which anomaly, though not usually noticed, I have repeatedly met with, and uniformly in concurrence with regular measles elsewhere on the body.

By some writers it is declared, that the exantheme may extend to all the passages of the interior to which the atmosphere has access. They describe it on the gums, and throughout the mouth and fauces, and on the surface of the pharynx, œsophagus, larynx, and trachea; and, by one of them, as even on the occluded contents of the thoracic and abdominal cavities. But the latter clause of the statement I entirely distrust. That it may exist in the throat, I am satisfied from my own observations, and can discern no reason why it might not be in the windpipe. May not, indeed, the croupy affection incident to the second or more advanced stage of the disease be caused by it?

The eruption retains its redness, which I have said is seldom or never of a deep hue, for two or three days, and then assumes a fainter colour, when it gradually vanishes altogether, and is followed by a meally or branny desquamation. But a peculiarity has been indicated with regard to the termination of the eruption, to which I must advert. It sometimes happens, that about the seventh or eighth day, the rash becomes livid, with a mixture of yellow, continuing for ten days or longer. This is the rubeola nigra of Willan, by whom, I believe, it was first remarked, though not as an alarming occurrence.

The eruption, in every variety of the disease, is rarely followed by an immediate abatement of the febrile symptoms. An exacerbation, indeed, oftener takes place, manifested by an increase of the pulse, heat of surface, more embarrassed respiration, cough, and soreness of the chest and abdomen, by greater turgescency of countenance, headach, or stupor, and in children especially, by a croupy hoarseness or watery diarrhœa. The nausea, vomiting, and other gastric or præcordial distress, usually cease as soon as the eruptive stage is over. Mostly the fever continues in some degree until the desquamation is completed, and may even to a later period. Yet the cough which forms such a prominent symptom, is ordinarily still more protracted,—lingering, at times, with great obstinacy. In some cases, so violent is the thoracic affection, as to amount to bronchitis, or pleurisy, or peripneumony, and croup has been noticed frequently in children. These may take place in any stage, and especially by an imprudent exposure to cold about the period of convalescence.

Measles is more purely inflammatory than any of its affiliated affections, to which, however, as to all general rules, there are exceptions. On some occasions, it assumes a character directly the reverse, the fever being real typhoid, and may-be little short of typhus gravior, which form of the disease has been called *putrid measles*. It was first described by Watson, and usually prevails as an epidemic. The most remarkable instances of its occurrence of which I have read, were at Plymouth in 1745, in London in 1763, and at Edinburgh in 1816. Extreme debility is represented early to supervene, with restlessness,—a constant propensity to vomit, and much disturbance of the cerebral functions,—sometimes fierce delirium, though more commonly coma, a dry, hard, or loaded and black tongue, swelled and mahogany coloured fauces, with an imperfect eruption, of a livid complexion, so much disposed to recession, that it takes place several times in the twenty-four hours.

As has been conjectured, it is not improbable, that the disease to which this description applies, was really scarlatina, having in regard to the throat affection especially, a greater resemblance than to measles. That the latter may assume the congestive state, it is, however, neither unreasonable to suppose, nor wanting in positive proof of its actually occurring. But it was chiefly distinguished as I have seen it, by a colder and paler collapsed skin, more gastric and cerebral disorder, by a greater sense of oppression, a less perfect developement of the eruption, and which was of fainter hue than ordinary, with an almost irresistible tendency to recede. Cases of it, I have indeed sometimes met with in which there was little or no catarrhal or other pulmonary affection, the alimentary canal and brain seeming exclusively to suffer, and here, constant vomiting, or purging, or both, as in cholera, with stupor and low delirium, and a purplish or livid eruption, interspersed by petechiæ and vibices, were the prominent characteristics.

There is a further modification of the disease, or so it is considered by some, termed *rubeola sine catarrho*, to which attention was first called by Willan. It occasionally prevails in this country, and is recognised by the familiar title of French measles. Characterized by the general symptoms of genuine measles, it differs from it in comparative mildness, the absence of catarrhal affections, as its name imports, the earlier appearance of the eruption, which is diffused in specks over the surface, and not arranged in a succession of definite crescents, and by a more transient continuance, usually subsiding in twenty-four hours. It may exist separately or in conjunction with common measles, and I have seen an attack of it succeeded by one of the latter in the course of eight or ten days. Being admitted to afford no protection in this respect, it is probably an efflorescence of another nature, dependent on some very different cause, or if at all of a morbillous character, it is illegitimate, and in this view is aptly called *rubeola spuria*.

What were the circumstances under which measles was generated, we know as little as of the origin of its kindred affections, and scarcely more of the cause of its periodical visitations. It sometimes occurs sporadically or sparsely, more commonly as an epidemic, "breaking out at the beginning of winter, increasing till the vernal equinox, and dying away towards the summer solstice,"*—though no season escapes, having seen it thus to occur in summer, and it is often the precursor or concomitant of variola or scarlatina, or some other of the exanthematous fevers.

It has been maintained, that it returns regularly once in seven years, and in support of the opinion facts are adduced. As a result of a thorough research into the subject, Professor Caldwell, now of Louisville, affirms that, beginning at the year 1772, and passing down to a period including fifty years, it prevailed epidemically in this city and vicinity, about every sixth year. How far this statement is correct, or on what data it is founded, I have no means of determining. Certain it is, however, as regards the later portion of this series of time, the fact has been otherwise. During the last thirty-five years, I do not think that we have had an exemption, for any long interval, from the disease. It may have been suspended for a year or more, though almost annually it might be met with, either sporadically or generally.

Excepting influenza, measles spreads perhaps more rapidly and diffusively in some instances, than any other epidemic. Not to cite other evidence of it, in the year 1801, in a few months, it overran nearly the whole of the United States, and in 1823, was scarcely less pervading. Nor is it improbable, that in such instances, its influence is extended to the brute creation. During its prevalence just alluded to, it is said, many of the domestic animals suffered severely from fever with catarrhal defluxions. But though thus observant of the epidemic character, there is still sufficient reason to suppose, that the immediate cause of the disease is a specific contagion, proving more or less operative, according to the constitution of the season, in this respect conforming to other analogous affections. Let me repeat here, that because a disease is an epidemic it is not necessarily uncontagious; in proof of which, among other instances, small-pox, the most conspicuously dependent on contagion, very frequently assumes this character, spreading rapidly and widely.

Existing epidemically, measles, it would appear, may be taken without any communication with the sick, or breathing the air of a room contaminated by such having recently occupied it. The atmosphere, seems to a great extent, to be infected during

* Sydenham.

the wide prevalence of the disease, and the inhalation of it any where within the vitiated limits, may adequately operate to this end. Facts are wanting to show decisively whether it can be disseminated by families, though I can scarcely doubt it. Nor is it better ascertained, at what stage of the disease the contagious property is evolved, or when it attains its greatest activity. Most probably, in each particular, it is subsequent to the eruption.

Notwithstanding the evidence to the contrary, I cannot think it one of those affections capable of propagation by inoculation. Experiments, I am aware, are appealed to, instituted by Home, then Professor at Edinburgh, who asserted, that inoculation succeeded, with great certainty, and produced a milder disease. This he effected by an application of cotton dipped in the blood of a patient with measles, to a scratch or slight incision in the skin. The fever, we are told, followed in six days,—was comparatively light, and without any disorder of the pulmonary organs. These experiments, however, seem never to have been confirmed; and their accuracy is questioned by most writers, and pointedly by Cazenave, in his late work on cutaneous diseases. By Willan it is reported, that he inoculated three children with the fluid of the vesicles, without any effect. But he cites the testimony of a Mr. Wachsel, who says that he succeeded in several instances where the operation was performed in the same mode.

To this point such is the only proof so far as I know, and my researches have not been limited. That it is not satisfactory, may be made, I think, very readily to appear. Could inoculation be practised with the certainty, and the beneficial effect attained in the mitigation of the disease, which are alleged, why, I demand, has not such an expedient been universally adopted, as was formerly the case with small-pox? Does not this fact alone, sufficiently invalidate the averments on the subject? But further, we learn that Thurmen and Tilligen utterly failed in their trials; and it is understood, that experiments were instituted in our Dispensary in 1801, in which the blood, the tears, the mucus of the nostrils and bronchiæ, the eruptive matter on the cuticle, properly moistened, were all tried without the least success. It is not unlikely, that in the few instances of alleged success by inoculation, the individuals had been previously exposed to the infection of the disease, and to this mode may its production be properly ascribed, the coincidence being mistaken for the effect, one of the most common sources of vitiation of our medical inductions.

The common opinion is, that the latent period of the morbillous contagion is eight days, or in other words, that the disease breaks out after an exposure to its cause, in that time, though on this point there is not unanimity,—Willan, for instance, making it from ten to fourteen days. Careful and extensive observations have satisfied me that the last is the incubative period, in this, too, among some other particulars, resembling small-pox.

Can measles be had a second time? That it is contrary to the tenor of the disease, is abundantly established. Yet anomalous cases do occur, among the most authentic of which, an account is given by the celebrated Baillie, of eight persons in the same family. In 1823, as prelusive to the varioloid epidemic, measles prevailed of a very malignant kind, very extensively in this city, and on that occasion several repetitions of it were observed. Failures of protection, however, may, perhaps, to a certain extent, be ascribed to the former attacks being of that variety of the disease entitled *rubeola sine catarrho*, which does not impair the susceptibility to genuine measles. Nevertheless, Willan and others have remarked, that under circumstances of epidemic prevalence, it is not uncommon for those who previously have had the genuine disease to be seized by a rubeolous fever, without any, or at all events, a very slight eruption. It, on the whole, may be, in this respect, assimilated to small-pox.

Not a little curious is it, that measles takes so firm a possession of the system, that its peculiar action is not readily supplanted by diseases apparently more violent, in proof of which, the case of small-pox may be adduced. Generally it has happened, that where the two affections co-existed, measles in a short time acquired the ascendency, and after running its course, small-pox was redeveloped. Examples, however, are recorded by Russel, of the two diseases passing through their regular stages, without impairing the force, or in any way affecting or modifying each other. But the most striking instances, perhaps, are those related by Dr. King, who tells us, that he inoculated with variolous matter forty-three children in the Foundling Hospital at Dublin, of whom sixteen sickened with measles, four or five days afterwards, and the small-pox appeared in due season, without apparently being at all influenced by the circumstance.

(To be continued.)

